# A tricky and rare cause of pulmonary eosinophilia: myeloid/lymphoid neoplasm with eosinophilia and rearrangement of PDGFRA

**DOI:** 10.1186/s12890-019-0967-7

**Published:** 2019-11-19

**Authors:** Magda Zanelli, Maxwell Smith, Maurizio Zizzo, Angelo Carloni, Riccardo Valli, Loredana De Marco, Moira Foroni, Andrea Palicelli, Giovanni Martino, Stefano Ascani

**Affiliations:** 1 Pathology Unit, Azienda Unità Sanitaria Locale-IRCCS di Reggio Emilia, Reggio Emilia, Italy; 2 Department of Pathology and Laboratory Medicine, Mayo Clinic, Scottsdale, AZ USA; 3 Surgical Oncology Unit, Azienda Unità Sanitaria Locale-IRCCS di Reggio Emilia, Reggio Emilia, Italy; 4 Clinical and Experimental Medicine PhD Program, University of Modena and Reggio Emilia, Modena, Italy; 5 Radiology Unit, Ospedale di Terni, University of Perugia, Perugia, Italy; 6 Hematology Unit, CREO, University of Perugia, Perugia, Italy; 7 Pathology Unit, Ospedale di Terni, University of Perugia, Perugia, Italy

**Keywords:** Eosinophilia, Myeloid, Lymphoid, Neoplasm, Lung, PDGFRA

## Abstract

**Background:**

Eosinophilic lung diseases represent a heterogeneous group of disorders with prominent infiltrate of eosinophils in lung interstitium and alveolar spaces. Peripheral blood eosinophilia is often present. Infections, drugs, allergens, toxic agents have to be evaluated as possible causes of eosinophilic lung infiltrates. The category of myeloid/lymphoid neoplasms with eosinophilia and rearrangement of PDGFRA, PDGFRB, FGFR1 and PCM1-JAK2 represents an uncommon cause of eosinophilic lung infiltrate.

**Case presentation:**

We report the case of a 70-year old man complaining of dry cough and dyspnea. Ground glass-opacities were seen on imaging studies and peripheral blood eosinophilia was present. A thorough step-wise patient’s evaluation led to identify the clonal nature of eosinophilia and the diagnosis of myeloid/lymphoid neoplasm with eosinophilia and rearrangement of PDGFRA was made.

**Conclusions:**

Correlation with clinical history, laboratory tests and imaging studies is essential to achieve the correct diagnosis when facing with eosinophilic lung infiltrates. A prolonged eosinophilia can cause life-threatening organ damage. Identification of PDGFRA rearrangement, as in the present case, is particularly critical given the sensitivity and excellent response to imatinib, which has completely changed the natural history of this neoplasm.

## Background

Eosinophilia comprises a heterogeneous group of disorders that, except for the eosinophilia feature itself, have few things in common [1]. As eosinophils can be found in different clinical settings, a careful investigation is essential to get to the correct diagnosis and adequate treatment [1].

Eosinophilia is more often secondary to a broad variety of both non-neoplastic and neoplastic disorders [1–6]. Clonal eosinophilia can be present in different hematological malignancies [1–6]. It is crucial to recognize and treat the underlying cause of eosinophilia. Patients with prolonged and marked eosinophilia are at risk of severe multiorgan damage related to eosinophil granules release [1–6]. The refractile eosinophilic granules contain major basic protein, eosinophil peroxidase and eosinophil cationic protein, substances important for eosinophil function in infection defense, immunomodulation and tissue inflammation [7].

The present challenging case of eosinophilia clarifies the progressive workup, which led to the diagnosis of myeloid/lymphoid neoplasm with eosinophilia and rearrangement of PDGFRA, a rare disease with less than 1 case per 1000000 persons per year [8]. In the present case the PDGFRA-rearranged neoplasm sustaining eosinophilia was effectively treated with imatinib with complete remission.

## Case presentation

**Fig. 1:**
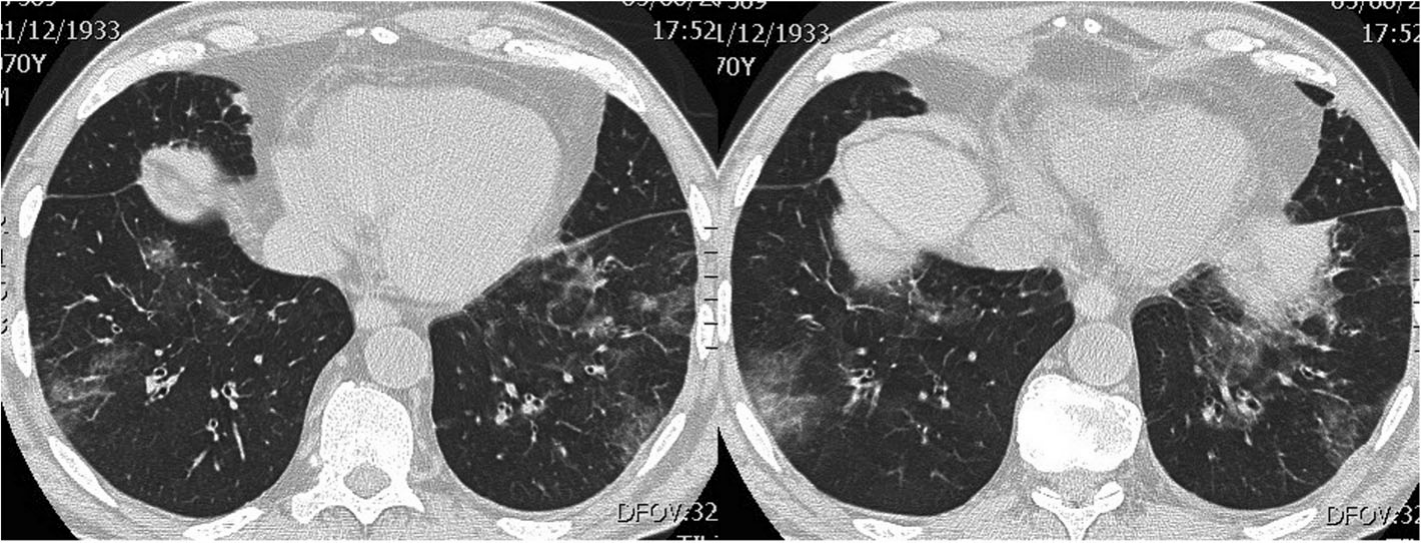
Axial HRCT scan of the chest revealed patchy ground-glass opacities bilaterally, predominantly in the lower lobes of the lungs

**Fig. 2:**
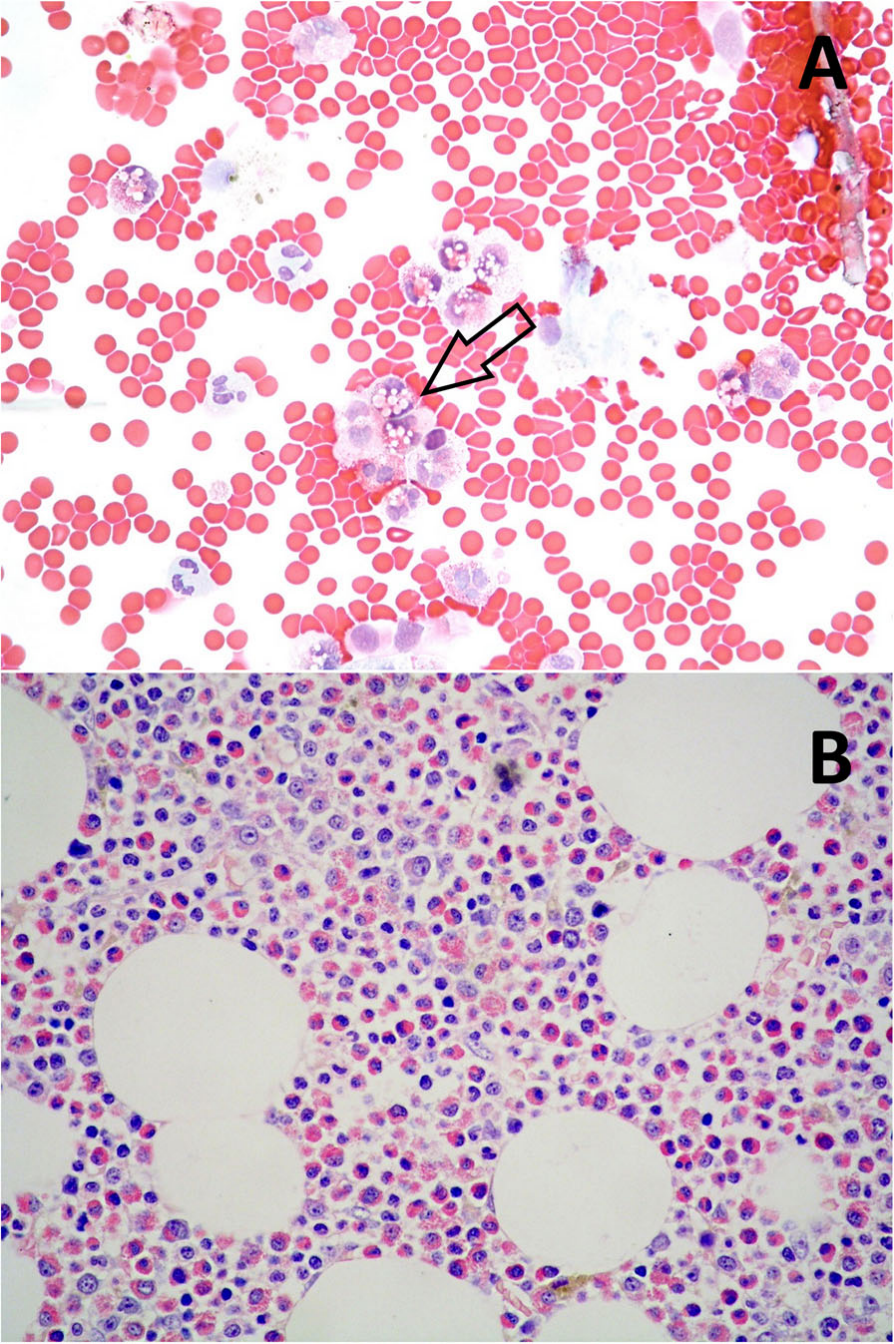
**a** Bronchialveolar fluid showing an increase in eosinophils, some of which degranulated and with cytoplasmic vacuoles (arrow) (magnification HE 40x); (**b**) Trephine biopsy section revealing an hypercellular and disorganized marrow with predominance of eosinophils and eosinophil precursors (magnification HE 40x)

**Fig. 3:**
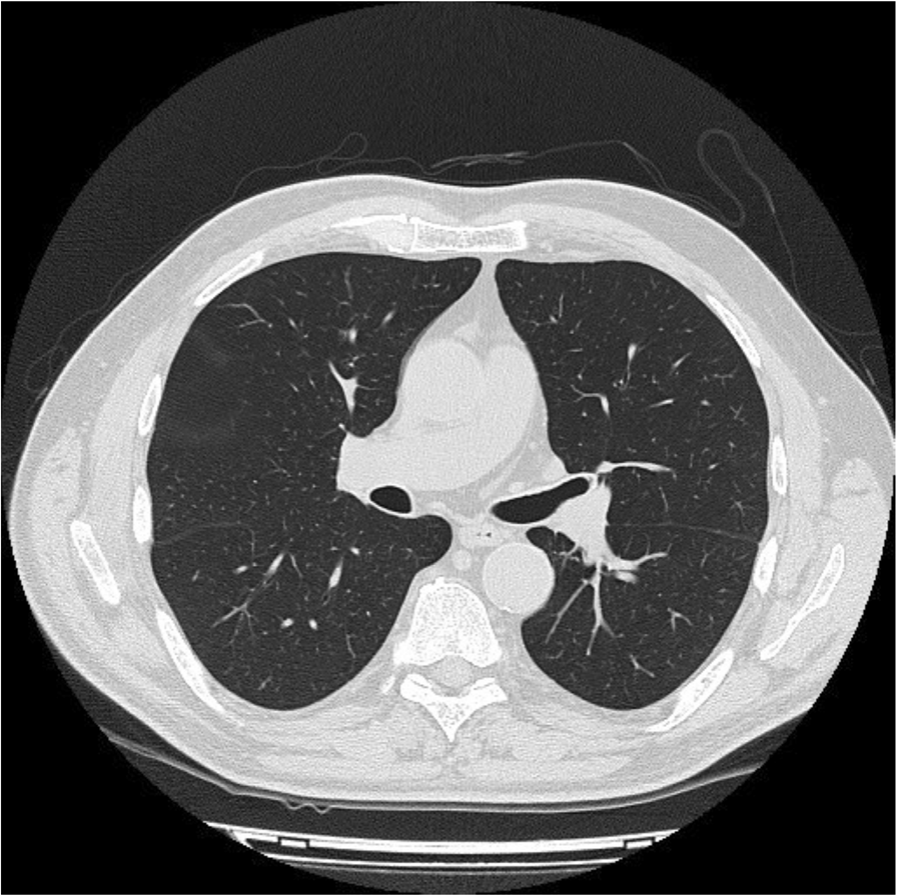
Axial HRCT scan of the chest showing complete radiological pulmonary resolution at 3 years from diagnosis

A 70-year old man presented with dry cough and dyspnea on exertion over the preceding 8 months. He was afebrile, with no history of allergies, asthma, drug intake or travelling. Physical examination revealed a moderately enlarged spleen; wheezes were present at pulmonary auscultation. Blood tests showed an increasing leukocytosis (17000/mmc) with up to 2000 eosinophils/mm3. Stool, urine and blood were negative for parasitic infections. Pulmonary function tests, with forced expiratory volume in 1 s (FEV1) of 60%, showed moderate small airway obstruction. High resolution computed tomography (HRCT) scan of the thorax revealed patchy ground-glass opacities bilaterally, predominantly in the lower pulmonary lobes (Fig. 1). Bronchioalveolar lavage (BAL) showed increased eosinophil percentage up to 60% of cells; most eosinophils appeared degranulated, with cytoplasmic vacuoles (Fig. 2a). Bone marrow aspirate showed numerous eosinophils and bone marrow trephine sections (Fig. 2B) revealed an hypercellular marrow with markedly increased eosinophils in different stages of maturation, including features of hypogranulation and nuclear hypersegmentation or hyposegmentation. No increase in mast cells was noted. The spectrum of eosinophil maturation raised concern for a myeloid neoplasm with eosinophilia. Fluorescence in situ hybridization (FISH) analysis was carried out and the fusion gene FIPL1-PDGFRA, occurring as a result of a cryptic deletion at 4q12, was identified. A conclusive diagnosis of myeloid/lymphoid neoplasm with eosinophilia associated with PDGFRA rearrangement was rendered. The patient received imatinib (100 mg daily), achieving a complete clinical, radiological (Fig. 3) and molecular remission at 3 years from diagnosis.

## Discussion and conclusions

Eosinophilia is defined as a peripheral blood eosinophil count greater than 1500/mm3 [1–6]. It can be secondary, representing a reactive response to different insults. In other cases eosinophilia is primary and the eosinophils themselves are neoplastic [1–6]. Eosinophilic infiltrates in the lungs with or without blood eosinophilia can be either secondary to infections (parasites, fungi, mycobacteria), allergens, drugs, toxic agents, connective vascular diseases, sarcoidosis or idiopathic (simple pulmonary eosinophilia/Löeffler syndrome, acute eosinophilic pneumonia, chronic eosinophilic pneumonia) [9]. Neoplasms associated with primary eosinophilia include: chronic myeloid leukemia (CML); acute myeloid leukemia (AML) associated with inversion 16 and translocation t(8;21); chronic eosinophilic leukemia, not otherwise specified (CEL-NOS); myeloid/lymphoid neoplasms with eosinophilia and rearrangement of PDGFRA, PDGFRB, FGFR1 and PCM1-JAK2 [1–6]. The PDGFRA-rearranged neoplasm is a rare disease with less than 1 case per 1000000 persons per year [8]. The above-mentioned neoplasms are all associated with primary eosinophilia, meaning that the eosinophils themselves are part of the clonal disorder. Differently systemic mastocytosis (SM) and lymphoid neoplasms (T-cell lymphomas and Hodgkin lymphoma) can cause secondary eosinophilia through cytokines production; eosinophils are not part of the neoplastic clone in these disorders, but are reactive in nature [1–6]. If the underlying cause of eosinophilia remains unknown after complete workup, hypereosinophilic idiopathic syndrome represents the diagnosis of exclusion.

The interest of our case resides mainly on the progressive thorough step-wise patient’s study leading to diagnosis and adequate treatment. The clinical presentation together with radiological pulmonary findings led to think of an interstitial lung disease. BAL evaluation identified a high percentage of eosinophils. In BAL specimens eosinophils are frequently degranulated or show cytoplasmic vacuolization and may be overlooked and misinterpreted either as neutrophils or macrophages [10]. In the normal population, eosinophils represent less than 1% of cells in BAL [10]. Eosinophilia is defined as more than 5% eosinophils, while severe eosinophilia is defined as more than 25% eosinophils [10]. An increased eosinophil count in BAL may be seen in asthma, drug reactions, infections, toxic agents, interstitial lung disease and connective tissue disorders [10].

After secondary causes of eosinophilia were excluded, our patient’s work-up proceeded to evaluate a primary bone marrow disorder. Bone marrow aspirate and trephine biopsy examination in conjunction with FISH analysis identified the presence of PDGFRA rearrangement.

The category of myeloid/lymphoid neoplasms with eosinophilia and rearrangement of PDGFRA, PDGFRB, FGFR1 and PCM1-JAK2 includes a group of disorders characterized by an aberrant tyrosine kinase activity resulting either from a fusion gene or a mutation [1–6]. This group of disorders can present as chronic myeloproliferative neoplasms, acute myeloid leukemia, or lymphoblastic leukemia/lymphoma [1–6]. Eosinophilia, both in peripheral blood and tissues, is a common feature of this group of neoplasms [1–6]. Tissue involvement by eosinophils, which are part of the neoplastic process, with release of granules content can cause severe organ damage [1–7]. Patients often present with cardiac, pulmonary, cutaneous or gastrointestinal symptoms, related to tissue infiltration by eosinophils. Pulmonary involvement may give rise to fibrosis and radiographically mimic an interstitial lung disease, as in our case [1–6].

In conclusion, recognizing the category of myeloid/lymphoid neoplasms with eosinophilia is crucial for treatment [1–6]. The aberrant tyrosine kinase activity characterizing PDGFRA and PDGFRB rearrangements, makes these disorders exquisitely responsive to tyrosine kinase inhibitors as imatinib, often with excellent results, as in the present case [1–6]. To date for FGFR1-rearranged neoplasms stem cell transplant represents the only potentially curative option; ongoing studies on the use of pemigatinib, a potent inhibitor of FGFR1, are showing promising efficacy in this group of neoplasms [11].

## Data Availability

All the original data supporting our research are described in the Case presentation section and in the figures’ legends.

## Abbreviations

BAL: Bronchioalveolar lavage
FISH: Fluorescence in situ hybridization
HRCT: High resolution computed tomography
